# Sex-specific inequalities in the use of drug-coated balloons for small coronary artery disease: a report from the BASKET-SMALL 2 trial

**DOI:** 10.1007/s00392-023-02249-6

**Published:** 2023-07-26

**Authors:** Maria Rubini Gimenez, Bruno Scheller, Ahmed Farah, Marc-Alexander Ohlow, Norman Mangner, Daniel Weilenmann, Jochen Wöhrle, Florim Cuculi, Gregor Leibundgut, Sven Möbius-Winkler, Marco Cattaneo, Nicole Gilgen, Christoph Kaiser, Raban V. Jeger

**Affiliations:** 1 Department of Cardiology and Cardiovascular Research Institute Basel (CRIB), University Hospital Basel and University of Basel, Basel, Switzerland; 2 Department of Internal Medicine/Cardiology, Heart Center Leipzig at University of Leipzig and Leipzig Heart Institute, Leipzig, Germany; 3 Clinical and Experimental Interventional Cardiology, Saarland University, Homburg/Saar, Germany; 4 Department of Cardiology Central Clinic Bad Berka, Bad Berka, Germany; 5 Department of Internal Medicine and Cardiology, Herzzentrum Dresden, Technische Universitaet Dresden, Dresden, Germany; 6 Department of Cardiology, Cantonal Hospital St. Gallen, St. Gallen, Switzerland; 7 Department of Cardiology, University Hospital Ulm, Ulm, Germany; 8 Department of Cardiology, Cantonal Hospital Luzern, Luzern, Switzerland; 9 Department of Cardiology, Cantonal Hospital Baselland, Liestal, Switzerland; 10 Department of Cardiology, Friedrich-Schiller-University of Jena, Jena, Germany; 11 Department of Clinical Research, University Hospital Basel, Basel, Switzerland; 12 Department of Cardiology, Triemli Hospital, Zurich, Switzerland

**Keywords:** Sex inequalities, Drug-coated balloon, Small coronary artery disease

## Abstract

**Background and objectives:**

Recent data have established non-inferiority of drug-coated balloons (DCB) compared to drug-eluting stents (DES) for treatment of small-vessel coronary artery disease. Since coronary vessels in women might have anatomical and pathophysiological particularities, the safety of the DCB strategy among women compared to men needs to be assessed in more detail.

**Methods:**

In BASKET-SMALL 2, patients with de novo lesions in coronary vessels < 3 mm and an indication for percutaneous coronary intervention were randomly allocated (1:1) to DCB vs. DES after successful lesion preparation. The primary objective of the randomized trial was to establish non-inferiority of DCB vs. DES regarding major adverse cardiac events (MACE; i.e., cardiac death, non-fatal myocardial infarction, and target vessel revascularization) after 12 months. The aim of the current sub-analysis is to evaluate whether the DCB strategy is equally safe among women and men after 12 and 36 months.

**Results:**

Among 758 randomized patients, 382 were assigned to DCB (23% women) and 376 to DES (30% women). In general, women were older, had more often diabetes mellitus and renal insufficiency, and presented more often with an acute coronary syndrome, whereas men were more often smokers, had multivessel disease and a previous history of acute myocardial infarction, and received a treatment with a statin. After 3 years, the primary clinical end point was not significantly different between groups (13% women vs. 16% men, HR 0.82; 95% CI 0.52−1.30; *p* = 0.40). There was no interaction between sex and coronary intervention strategy regarding MACE at 36 months (10% women vs. 16% men in DCB, 16% women vs. 15% men in DES; *p*_interaction_ = 0.31).

**Conclusion:**

In small native coronary artery disease, there was no statistically significant effect of sex on the difference between DCB and DES regarding MACE up to 36 months.

**Clinical trial registration:**

URL: http://www.clinicaltrials.gov. Unique identifier: NCT01574534.

**Graphical abstract:**

**Figure:**
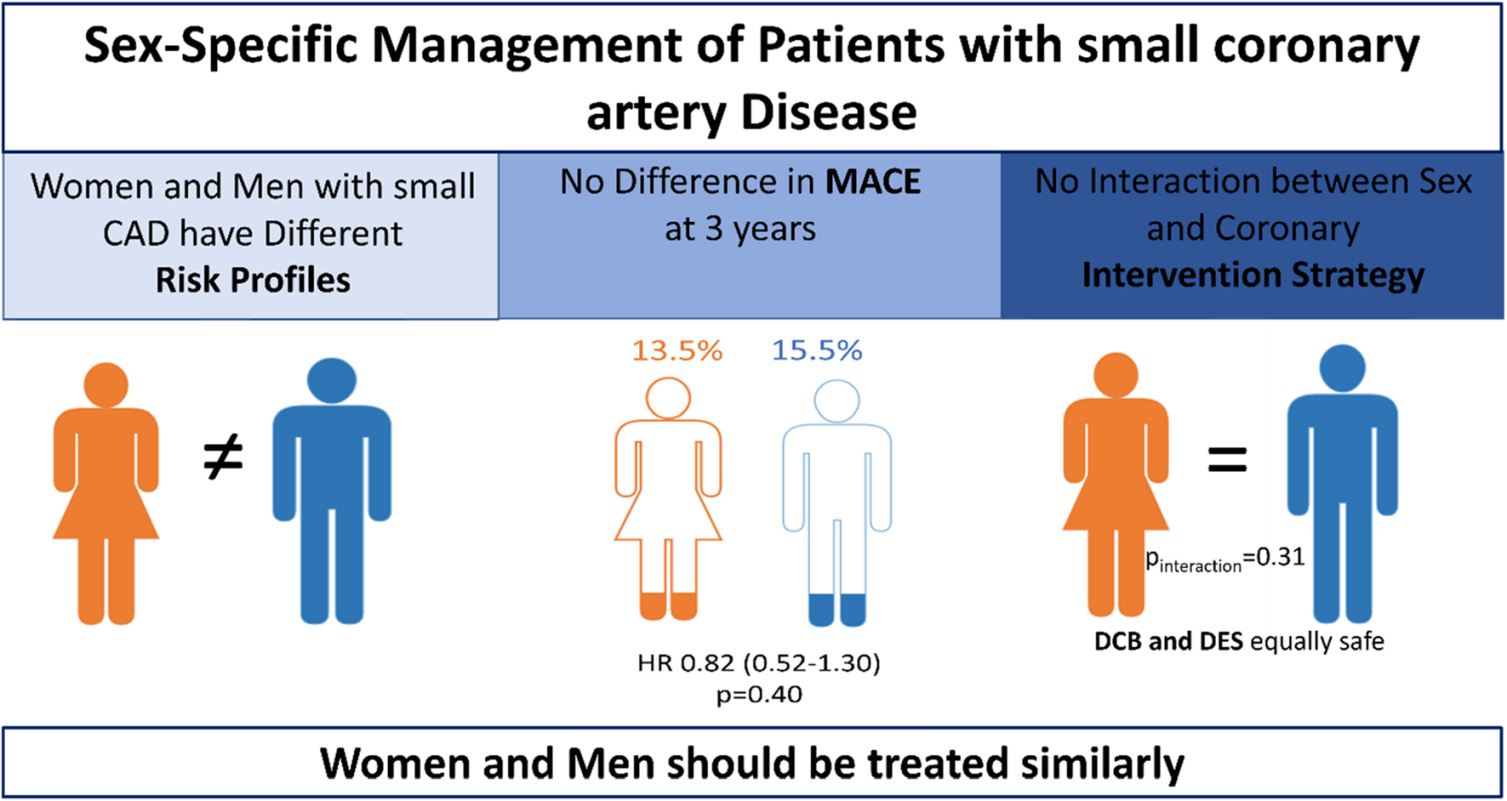
*CAD* coronary artery disease, *MACE* major adverse cardiovascular events, *HR* Hazard ratio, *DCB* drug-coated balloon, *DES* drug-eluting stent

**Supplementary Information:**

The online version contains supplementary material available at 10.1007/s00392-023-02249-6.

## Introduction

Female sex has been linked to a poorer prognosis after coronary revascularization. Women undergoing percutaneous coronary intervention (PCI) have a higher risk of death, myocardial infarction (MI), and other procedure-related complications, mainly due to older age, a higher prevalence of comorbidities, and a more severe coronary artery disease (CAD) risk profile [1–6]. 

Drug-coated balloons (DCB) are an established therapeutic option for restenosis of bare metal stents [7] and drug-eluting stents (DES) [8]. They recently showed short- and long-term non-inferiority compared to DES for treatment of small-vessel coronary artery diseases, suggesting that a stent-free technique with DCB only might be a safe strategy after appropriate lesion preparation [9, 10]. One prerequisite of the DCB-only technique in de novo stenoses is a careful preparation of the lesion without a residual stenosis of more than 30% or a flow-limiting dissection [11]. Over the last years, studies have shown the higher predisposition of women for spontaneous and post-dilatation coronary dissections, with hormones possibly playing a potential role in their pathophysiology [12–14]. Whether this higher risk for dissections might influence the feasibility of a DCB-only approach for small-vessel coronary artery disease among women and whether the strategy is equally safe among women and men is still unknown.

Thereby, we aim to assess in the randomized population of the BASKET-SMALL 2 trial whether a stent-free strategy with DCB only for small-vessel coronary disease is an equally safe strategy among women and men.

## Methods

### Trial design

This is a predefined subgroup analysis of the BASKET-SMALL 2 trial as prespecified in the study protocol. The prospective, randomized, multicenter open-label, non-inferiority BASKET-SMALL 2 trial [9, 10, 15] with 14 participating European centers included patients with de novo lesions (< 3 mm in diameter) in coronary vessels and an indication for PCI. Patients with successful lesion preparation were randomly allocated (1:1) to receive angioplasty with DCB versus implantation of a second-generation DES after randomization via an interactive Internet-based response system, while patients with a residual stenosis > 30% or flow-limiting dissections were treated with DES and entered a registry. Dual antiplatelet therapy was given according to current guidelines. Design details (including inclusion and exclusion criteria) and procedural details have been published previously and are shown in the online supplement (Online Supplement, Methods). The primary objective of the randomized trial was to establish non-inferiority of DCB vs. DES regarding major adverse cardiac events (MACE; i.e., cardiac death, non-fatal myocardial infarction, and target vessel revascularization) after 12 months. For the current analysis, outcome was assessed up to 36 months, and results were analyzed according to sex.

### Statistical analysis

Statistical analyses were performed on the trial data set of randomized patients and the registry according to the intention-to-treat principle, i.e., all trial patients were analyzed on the basis of the treatment they were randomly allocated to. All analyses were conducted with the statistical software package R [16], using “two-sided” statistical tests and confidence intervals. No correction for multiple testing was applied and missing data were handled through available case analyses. Categorical data are presented as frequencies and percentages (with the difference between study arms analyzed by Pearson’s Chi-squared test). For numerical variables, the mean and standard deviation, or the median and interquartile range are presented, as appropriate, with the difference between study arms analyzed by Student’s t test or Wilcoxon–Mann–Whitney test, respectively.

For each end point, treatment and covariates’ effects on the times to event were tested by Cox regressions, with study center as a stratifying factor to account for differences in baseline hazards. The Kaplan–Meier estimates of the event rates are reported along with the corresponding maximum-likelihood hazard ratios (HR) estimates and 95% Wald confidence intervals (CI). The assumptions of proportional hazards and homogeneity of treatment effects among study centers in the Cox models were checked by testing the correlation of the scaled Schoenfeld residuals with time and the interaction of the stratifying factor study center with treatment in the Cox models, respectively. The end points of patients not experiencing an event were considered as censored on the last observation date.

## Results

### Patient characteristics

The study population consisted of a total of 882 patients eligible for assessment, of which 758 (86%) were included in the randomized trial and 124 (14%) entered the separate registry. Of the 124 patients in the registry, 25 (20%) were women.

**Fig. 1:**
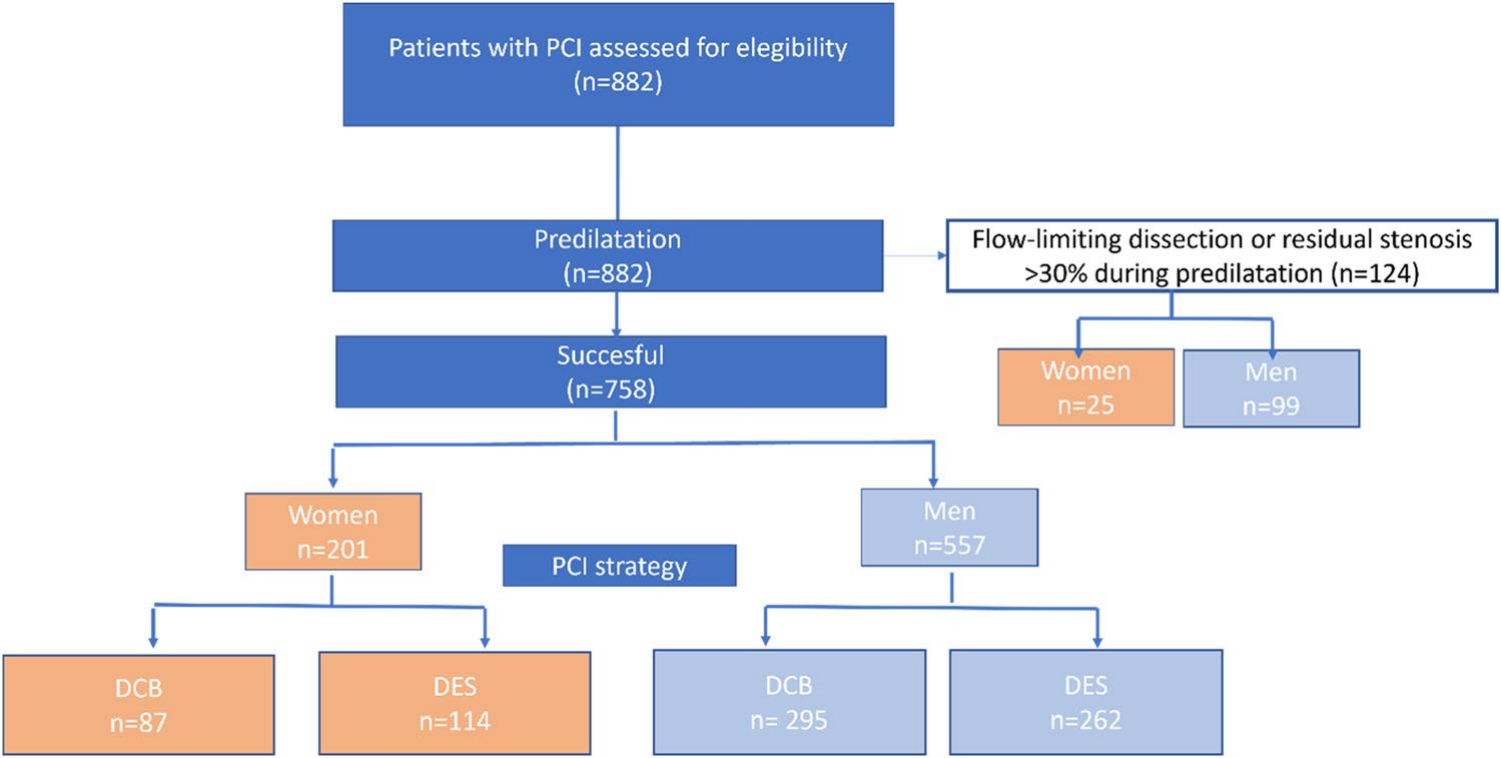
Study flow. Flowchart displaying women and men randomized in the study

Of the 758 patients in the randomized trial, 382 were assigned to the DCB group (23% women) and 376 to the DES group (30% women, Fig. 1). Compared to men, women were older and had more often diabetes mellitus and renal insufficiency, whereas men were more often smokers, and had more often a previous history of acute myocardial infarction, PCI, and a treatment with statin (Table 1).

**Table 1:** Baseline characteristics

	All	Women	Men	*p* value
	*n* = 758	*n* = 201	*n* = 557	
Age—years				
Mean (SD)	67.8 (10.3)	70.0 (10.3)	67.0 (10.3)	** < 0.001**
BMI kg/m^2^				
Mean (SD)	28.3 (4.5)	28.5 (5.2)	28.2 (4.3)	0.35
Cardiovascular risk factors *n* (%)				
Current smoking	154 (20.8)	34 (17.4)	120 (22.0)	0.22
Hypertension	656 (86.8)	182 (90.5)	474 (85.4)	0.08
Hypercholesterolemia	521 (69.4)	139 (69.8)	382 (69.2)	0.055
Diabetes mellitus				**0.002**
IDDM	95 (12.6)	39 (19.4)	56 (10.1)	
NIDDM	157 (20.8)	35 (17.4)	122 (22.1)	
Previous myocardial infarction *n* (%)	293 (38.7)	64 (31.8)	229 (41.1)	**0.026**
Previous PCI *n* (%)	476 (62.8)	113 (56.2)	363 (66.2)	**0.030**
Previous stroke *n* (%)	39 (5.2)	10 (5.0)	20 (30.6)	1.00
Known peripheral artery disease *n* (%)	53 (7.0)	15 (7.5)	38 (6.8)	0.89
Renal dysfunction *n* (%)	174 (23.0)	62 (30.8)	112 (20.1)	**0.027**
Previous therapy *n* (%)				
Clopidogrel	205 (27.0)	46 (22.9)	159 (28.5)	0.15
Aspirin	611 (80.6)	156 (77.6)	455 (81.7)	0.25
Prasugrel	74 (9.8)	14 (7.0)	60 (10.8)	0.16
Ticagrelor	118 (15.6)	35 (17.4)	83 (14.9)	0.46
Anticoagulants	64 (8.7)	16 (8.2)	48 (8.9)	0.87
Statin	502 (66.3)	120 (59.7)	382 (68.7)	**0.026**
Left ventricular ejection fraction-%				
Median (IQR)	60 (53–62)	60 (55–62)	60 (51–62)	0.39

Bold is to highlight statistical significance

*IQR* interquartile range, *BMI* body mass index, *IDDM* insulin-dependent diabetes mellitus, *NIDDM* non-insulin-dependent diabetes mellitus, *PCI* percutaneous coronary intervention

### Procedural findings

Procedural characteristics were similar among women and men. However, women presented more often with an acute coronary syndrome (ACS) rather than a chronic coronary syndrome, as well as a bifurcation lesion, whereas among men the right coronary artery was more often the vessel elected for PCI. In addition, men presented more often with a multivessel coronary disease, and required a higher number and length of DCB or DES (Tables 1 and 2). Duration of dual antiplatelet therapy did not differ between the different groups (Supplemental Fig. 1S).

**Table 2:** Procedural characteristics

	All	Women	Men	*p* value
	*N* = 758	*n* = 201	*N* = 557	
Presentation				
Acute coronary disease *n* (%)	214 (28.2)	69 (34.3)	145 (26.0)	**0.032**
Chronic coronary syndrome *n* (%)	544 (71.8)	132 (65.7)	412 (74.0)	**0.032**
Target vessel for PCI *n* (%)				**0.017**
Left anterior descending	244 (32.2)	162 (29)	82 (41)	
Left circumflex	362 (47.8)	280 (50)	82 (41)	
Right coronary	152 (20.0)	115 (21)	37 (18)	
Multivessel coronary disease *n* (%)	598 (78.9)	147 (73.1)	451 (81.0)	**0.026**
Bifurcation lesion *n* (%)	51 (6.9)	21 (10.8)	30 (5.5)	**0.019**
Procedural success				
Mean (SD)	0.97 (0.2)	0.97 (0.2)	0.97 (0.2)	0.67
Number of DCB or DES				
Mean (SD)	1.2 (0.6)	1.15 (0.4)	1.27 (0.6)	**0.013**
Length of DCB or DES				
Mean (SD)	19.0 (5.43)	18.08 (4.76)	19.47 (5.6)	**0.002**
Effective Size of DCB or DES				
Mean (SD)	2.5 (0.27)	2.52 (0.27)	2.54 (0.3)	0.60
Inflation pressure				
Mean (SD)	12.1 (3.35)	12.02 (3.2)	12.06 (3.4)	0.90
Compliant balloon *n* (%)	558 (73.6)	157 (78.1)	401 (72.0)	0.11

Bold is to highlight statistical significance

### Clinical end point at 1 and 3 years

**Fig. 2:**
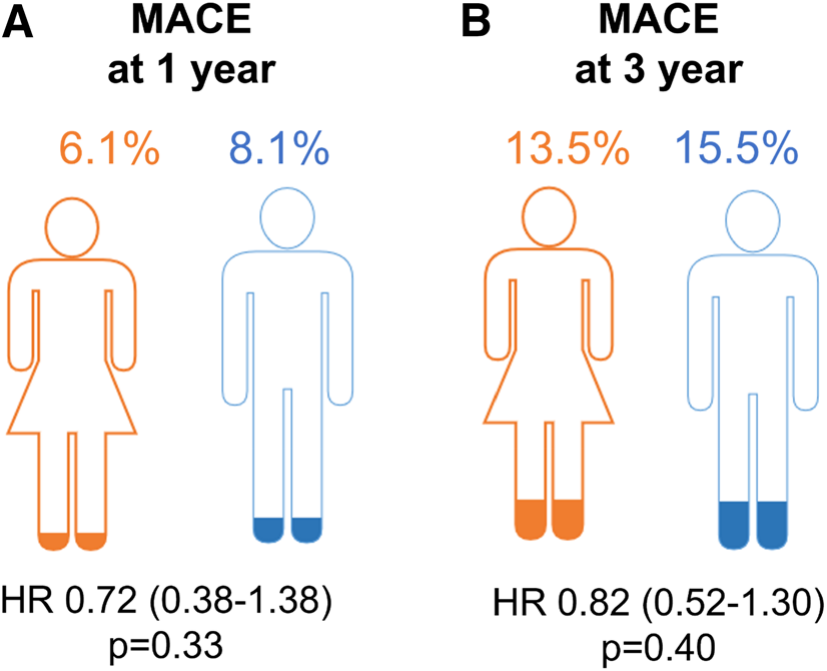
MACE at 1 and 3 years. Hazard ratios in women vs. men for MACE **A** at 1 year and **B** at 3 years

**Fig. 3:**
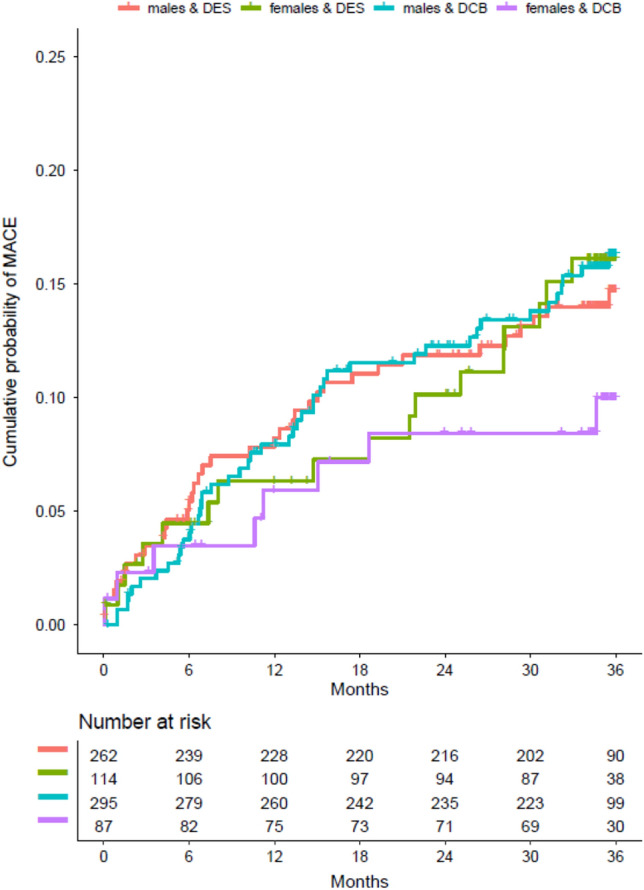
Time to primary end point. Kaplan–Meier estimates time-to-event curves for the primary end point (composite of cardiac death, non-fatal myocardial infarction, and target vessel revascularization) during 3 years according to sex and PCI strategy

After 1 year, MACE was not significantly different between groups (6% women versus 8% men, HR 0.72; 95% CI 0.38–1.38; *p* = 0.33, Figs. 2 and 3). There was no interaction between sex and coronary intervention strategy regarding MACE (*p*_interaction_ = 0.91), while the primary end point occurred in 6% of women vs. 8% men treated with either strategy. After 3 years, there were no significant differences neither (13% women vs. 16% men, HR 0.82; 95% CI 0.52–1.30; *p* = 0.40) (Figs. 2 and 3). Similarly, no differences were observed for the single events which were included in the definition of MACE (Supplemental Figs. 2S, 3S, 4S).

Similarly, when looking at the effect of each variable on the hazard of 3-year MACE and its interaction with sex, no significant interactions were observed. (Supplemental Table 1S).

The 3-year follow-up was completed by 516/557 (92.6%) men and 178/201 (88.6%) women. The 1-year follow-up was completed by 524/557 (94.1%) men and 188/201 (93.5%) women.

## Discussion

The present predefined subgroup analysis of the randomized BASKET-SMALL 2 trial investigating sex-specific outcomes can be summarized as follows. First, women and men eligible for randomization had different baseline characteristics with a higher risk profile, but less known coronary artery disease in women. Second, women and men had similar outcomes with respect to mortality, recurrent MI, and target vessel revascularization (TVR) after 1 and 3 years. Third, women suffering from these outcomes had a similar profile compared to women without. Fourth, sex did not influence the main findings of the trial showing non-inferiority of the DCB strategy.

Our data corroborates and extends previous work in the field of sex differences in coronary interventions. Early studies have shown poorer outcome among women with CAD compared to men [17]. However, women with CAD are usually older and have therefore more comorbidities that might affect outcomes; many of these studies showed similar outcomes after adjustment for confounders, suggesting that these differences might be attributed to the confounders and not associated with sex [18, 19]. Similarly, our results seem to confirm data reported by more recent studies [6] where mortality and adverse events after coronary interventions were similar among women and men. Although in the present trial women and men presented with some baseline differences possibly suggesting a higher cardiovascular burden for women (i.e., older, more often diabetes mellitus, renal insufficiency, and presentation as an ACS), there were no differences regarding outcomes, even before adjustment. A DCB treatment has been safe and effective in those subgroups of patients with diabetes, renal insufficiency, high bleeding risk, and ACS [20–23]. The most probable explanation for this could be the fact that men presented with a more progressed and established CAD (previous history of AMI and PCI), despite having a lower cardiovascular risk profile. Nevertheless, recent data limited exclusively to patients with MI [24] showed that younger women (below 50 years) who experienced an MI had a worse outcome due to lower likelihood to undergo a coronary angiography. Contrarily, women who underwent a coronary intervention did not show a worse outcome compared to men, suggesting that the poorer outcome among those women suffering MI may reside in the gaps between diagnosis and treatment before PCI (maybe due to the lack of awareness of the seriousness of the disease) rather than the procedural success or the sex condition per se. This hypothesis would explain why in our results the only precondition for a worse outcome among women was the presentation as an ACS. Additionally, data for the optimal sex-specific coronary revascularization strategy were scarce until recently. In or study, no statistically significant difference was observed between women and men regarding the consequences of the coronary intervention strategy choice in terms of MACE up to 36 months. Subsequently, there is no significant sex effect on the difference between DCB and DES.

Finally, the pathomechanism of CAD shows some sex-related disparities. It is well known that the delayed development of CAD in women is in part due to estrogen protection and occurs almost 10 years later compared to men [25]. However, it has become increasingly apparent that multiple factors may predispose to an arteriopathy among women that can weaken the arterial wall and increase vulnerability for dissection [26, 27]. Our data suggest that DCB strategy work well in women as well as in men showing no differences in the number of eligible patients for randomization.

Some limitations merit being considered. First, the number of women included in the present study was lower compared to men. According to previous studies, women are still underrepresented in most of coronary intervention trials and the rate of enrolled women is still far from 50%. The reason for this underrepresentation is still unclear. It might be due to lower incidence in general terms of MI or to a underdiagnosis due to atypical symptoms, which leads to lower inclusion in trials. All these data seem to suggest that efforts need to be made to mitigate the sex misbalance in terms of enrolled patients. Second, there was no routine angiographic follow-up in the study; therefore, event rates as for example TVR could have been underestimated [21]. Third, it seems that there is a trend for a lower MACE in female treated with DCB, which may not be significant due to the small size of the sample. Finally, since the BASKET-SMALL 2 trial was designed to assess coronary interventions, we cannot comment on potential pre-angiography sex-specific differences (i.e., time to medical contact or awareness of severity).

In conclusion, although women eligible for a DCB-PCI presented with a different profile, major adverse event rates after 1 and 3 years were similar to those in men. These data suggest that a DCB strategy to treat small native coronary artery disease is similarly safe among women and men.

### Supplementary Information

Below is the link to the electronic supplementary material.

Supplementary file1 (DOCX 157 kb)

## Abbreviations

PCI: Percutaneous coronary intervention
MI: Myocardial infarction
CAD: Coronary artery disease
DCB: Drug-coated balloons
DES: Drug-eluting stents
TVR: Target vessel revascularization
