# Supplementary material for: Sex-specific inequalities in the use of drug-coated balloons for small coronary artery disease: a report from the BASKET-SMALL 2 trial
Source: Clin Res Cardiol. 2023 Jul 26;113(7):959–66. doi: 10.1007/s00392-023-02249-6 (PMC11219376; doi:10.1007/s00392-023-02249-6)
Supplement: Supplementary file 1 — Supplementary file1 (DOCX 157 kb) [file 392_2023_2249_MOESM1_ESM.docx]

**ONLINE SUPPLEMENTAL MATERIAL**

1. **Methods**
2. **Tables**
3. **Figures**
4. **Methods**

**Design and procedural details**

**Inclusion criteria**

Consecutive patients with an indication for PCI due to acute coronary syndrome, chronic angina or silent ischemia and:

1. Angiographic lesions in native coronary arteries with a diameter of 2mm to less than 3mm
2. Successful predilatation of the lesion with angioplastic balloon (no higher-grade dissection (only A and B), no higher-grade stenosis (<30%), no flow limitation)

**Exclusion Criteria**

1. concomitant PCI of large lesions of at least 3 mm in diameter in the same epicardial coronary artery
2. PCI of in-stent restenosis
3. life expectancy of less than 12 months
4. pregnancy
5. enrolment in another randomised trial for coronary intervention
6. inability to give informed consent

**Procedures**

Participants in the DCB group received the paclitaxel-coated balloon SeQuent Please (B Braun Melsungen AG, Melsungen, Germany), and those in the DES group received one of two second-generation DES: the everolimus-eluting Xience stent (Abbott Vascular, Santa Clara, CA, USA) or the paclitaxel-eluting Taxus Element stent (Boston Scientific, Natick, MA, USA). The study was started with Taxus Element as the comparator (to ensure that devices with similar agents were used), but later (between June 19, 2013, and Jan 24, 2014) had to be continued with Xience because the initial stent became unavailable. Specifically, the DCB, which had to be 2–3 mm longer on each side than the predilatation balloon, was inflated at nominal pressure for a minimal time of 30 s. In cases with flow-limiting dissections or residual angiographically significant stenosis (ie, >30% stenosis) after DCB treatment, additional spot stenting avoiding geographical mismatch was allowed. PCI was done under dual antiplatelet therapy with acetylsalicylic acid (100 mg per day) and either a thienopyridine (clopidogrel [75 mg per day] or prasugrel [10 mg per day]) or ticagrelor (90 mg twice per day). After PCI, dual antiplatelet therapy was continued in stable patients for 4 weeks (for DCB) or 6 months (for DES) and in patients with acute coronary syndrome for 12 months. Dual antiplatelet therapy was given for 3 months in patients treated with a combination of DCB and BMS, and for 6 months in patients with DCB and DES. In patients on oral anticoagulation, we followed the guidelines irrespective of DCB or DES treatment.

1. **Tables**

| **Table 1S** Effect of each variable on | the hazard of 3y-MACE and | its interaction with sex |  |
| --- | --- | --- | --- |
| **Risk factor** | **Variable** | **3y HR (95%CI)** | **p-value** |
| **Age** | Age: for each additional year | 1.00 (0.98-1.01) | 0.64 |
|  | Sex: women vs men | 0.83 (0.52-1.33) | 0.44 |
|  | Interaction: age & sex | 1.02 (0.98-1.07) | 0.40 |
| **BMI** | BMI: for each additional kg/m2 | 1.02 (0.98-1.07) | 0.28 |
|  | Sex: women vs men | 0.84 (0.53-1.33) | 0.46 |
|  | Interaction: BMI & sex | 0.97 (0.89-1.07) | 0.60 |
| **Current smoking** | smoking: yes vs no | 0.76 (0.45-1.30) | 0.32 |
|  | Sex: women vs men |  |  |
|  | interaction: smoking & sex | 0.66 (0.17-2.53) | 0.55 |
| **Hypertension** | hypertension: yes vs no | 1.11 (0.55- 2.23) | 0.78 |
|  | Sex: women vs men | 0.78 (0.48- 1.28) | 0.33 |
|  | interaction: hypertension & sex | 3.69 (0.45-30.18) | 0.22 |
| **Hypercholesterolaemia** | hypercholesterolaemia:yes vs no | 1.03 (0.66- 1.58) | 0.91 |
|  | Sex: women vs men | 0.83 (0.53- 1.32) | 0.44 |
|  | interaction: hypercholesterolaemia&sex | 0.71 (0.27-1.88) | 0.50 |
| **Family History** | family history: yes vs no | 1.07 (0.70- 1.62) | 0.76 |
|  | Sex: women vs men | 0.83 (0.51- 1.35) | 0.45 |
|  | interaction: family history & sex | 0.94 (0.36-2.48) | 0.90 |
| **Diabetes Mellitus** | diabetes: yes vs no | 1.91 (1.27- 2.86) | **0.002** |
|  | Sex: women vs men | 0.80 (0.49- 1.29) | 0.36 |
|  | interaction: diabetes & sex | 1.14 (0.46-2.82) | 0.77 |
| **Previous anterior MI** | prev. anterior MI: yes vs no | 1.93 (0.70-5.30) | 0.20 |
|  | Sex: women vs men | 0.79 (0.49- 1.27) | 0.33 |
|  | interaction:prev.anterior MI&sex | 1.93 (0.70-5.30) | 0.20 |
| **Previous any MI** | prev. any MI: yes vs no | 1.38 (0.93- 2.06) | 0.11 |
|  | Sex: women vs men | 0.87 (0.55- 1.37) | 0.54 |
|  | interaction: prev. any MI & sex | 0.78 (0.30-1.99) | 0.60 |
| **Previous PCI** | prev. PCI: yes vs no | 1.73 (1.09- 2.73) | 0.20 |
|  | Sex: women vs men | 0.91 (0.57- 1.45) | 0.68 |
|  | interaction: prev. PCI & sex | 0.71 (0.26-1.91) | 0.50 |
| **Heart Failure** | heart failure: yes vs no | 2.04 (1.21- 3.42) | **0.007** |
|  | Sex: women vs men | 0.76 (0.47- 1.24) | 0.27 |
|  | interaction: heart failure & sex | 1.76 (0.60-5.19) | 0.30 |
| **Renal disease** | renal disease: yes vs no | 1.49 (0.90- 2.45) | 0.12 |
|  | Sex: women vs men | 0.84 (0.53- 1.33) | 0.45 |
|  | interaction: renal disease & sex | 1.03 (0.33-3.22) | 0.97 |
| **COPD** | COPD: yes vs no | 1.93 (1.08- 3.46) | **0.027** |
|  | Sex: women vs men | 0.77 (0.48- 1.25) | 0.30 |
|  | interaction: COPD & sex | 1.88 (0.58-6.08) | 0.29 |
| **Rheumatologic disorder** | rheumatologic disorder:yes vs no | 2.01 (0.99- 4.07) | 0.05 |
|  | Sex: women vs men | 0.82 (0.51- 1.31) | 0.40 |
|  | interaction: rheuma & sex | 0.99 (0.23-4.32) | 0.99 |
| **Liver disease** | liver disease: yes vs no | 3.71 (1.41- 9.76) | 0.008 |
|  | Sex: women vs men | 0.84 (0.53- 1.33) | 0.45 |
|  | interaction: liver disease & sex | 0.40 (0.04-3.99) | 0.43 |
| **LV ejection fraction-%** | LVEF:for each additional %point | 0.98 (0.96- 1.00) | 0.10 |
|  | Sex: women vs men | 0.71 (0.41- 1.26) | 0.24 |
|  | interaction: LVEF & sex | 1.01 (0.95-1.06) | 0.84 |
| **Acute Coronary Syndrome** | ACS: yes vs no | 1.10 (0.70- 1.73) | 0.67 |
|  | Sex: women vs men | 0.80 (0.50- 1.30) | 0.37 |
|  | interaction: ACS & sex | 1.43 (0.55-3.72) | 0.46 |
| **Chronic Coronary Syndrome** | CCS: yes vs no | 0.91 (0.58- 1.42) | 0.67 |
|  | Sex: women vs men | 0.80 (0.50- 1.30) | 0.37 |
|  | interaction: CCS & sex | 0.70 (0.27-1.82) | 0.46 |
| **Multivessel coronary disease** | MVC disease: yes vs no | 1.65 (0.94- 2.89) | 0.08 |
|  | Sex: women vs men | 0.87 (0.55- 1.38) | 0.55 |
|  | interaction: MVS & sex | 0.83 (0.25-2.72) | 0.76 |

Hazard ratios (HR), confidence intervals (CI), and p-values from Cox regressions stratified by study center and treatment, adjusted for sex, and including the interaction of variable and sex.

| Table 2S | Individual MACE endpoints |  |  |  |  | |  |
| --- | --- | --- | --- | --- | --- | --- | --- |
| Type of event | **Subgroup** | **Study arm** | **1y-events (rate)** | **1y HR (95%CI) p-value** | **3y-events (rate)** | | **3y HR (95%CI) p-value** |
| MACE | Males | DES  DCB | 21 (8.2%)  23 (7.9%) | 1-reference  0.93 (0.52-1.69) p=0.82 | | 36 (14.7%)  45 (16.3%) | 1-reference  1.08 (0.70-1.68) p=0.72 |
| MACE | Females | DES  DCB | 7 (6.3%)  5 (5.9%) | 1-reference  1.20 (0.36-3.96) p=0.77 | | 17 (16.1%)  8 (10.0%) | 1-reference  0.68 (0.29-1.60) p= 0.38 |
| Cardiac death | Males | DES  DCB | 5 (2%)  8 (2.8%) | 1-reference  1.39 (0.46-4.26) p=0.56 | | 8 (3.5%)  13 (4.6%) | 1-reference  1.42 (0.59-3.42) p=0.44 |
| Cardiac death | Females | DES  DCB | 0 (0%)  4 (4.8%) | Perfect separation  Perfect separation | | 5 (4.9%)  4 (4.8%) | 1-reference  1.07 (0.28-4.03) p=0.92 |
| Non-fatal MI | Males | DES  DCB | 7 (2.7%)  5 (1.8%) | 1-reference  0.63 (0.20-1.98) p=0.42 | | 15 (6.1%)  16 (6.2%) | 1-reference  0.96 (0.47-1.94) p=0.91 |
| Non-fatal MI | Females | DES  DCB | 6 (5.4%)  1 (1.2%) | 1-reference  0.32 (0.04-2.75) p=0.30 | | 8 (7.5%)  3 (4.1%) | 1-reference  0.60 (0.15-2.35) p=0.56 |
| TVR | Males | DES  DCB | 13 (5.2%)  12 (4.2%) | 1-reference  0.78 (0.36-1.72) p=0.55 | | 24 (9.8%)  26 (9.8%) | 1-reference  0.94 (0.54-1.64) p=0.83 |
| TVR | Females | DES  DCB | 4 (3.6%)  1 (1.2%) | 1-reference  0.35 (0.04-3.18) p=0.35 | | 8 (7.7%)  4 (5.5%) | 1-reference  0.67 (0.20-2.24) p=0.51 |
| All-causes death | Males | DES  DCB | 7 (2.7%)  13 (4.5%) | 1-reference  1.59 (0.63-3.98) p=0.33 | | 17 (7.1%)  23 (8.1%) | 1-reference  1.17 (0.63-2.20) p=0.62 |
| All-causes death | Females | DES  DCB | 2 (1.8%)  4 (4.8%) | 1-reference  3.39 (0.53-21.58) p=0.20 | | 10 (9.2%)  5 (6.1%) | 1-reference  0.77 (0.26-2.29) p=0.64 |

MACE : major adverse cardiac events; HR: Hazard ratio; y: year; MI: myocardial infarction; TVR: target vessel revascularization; DES: drug-eluting stents; DCB: drug-coated balloons

1. **Figures**

**Figure 1S**


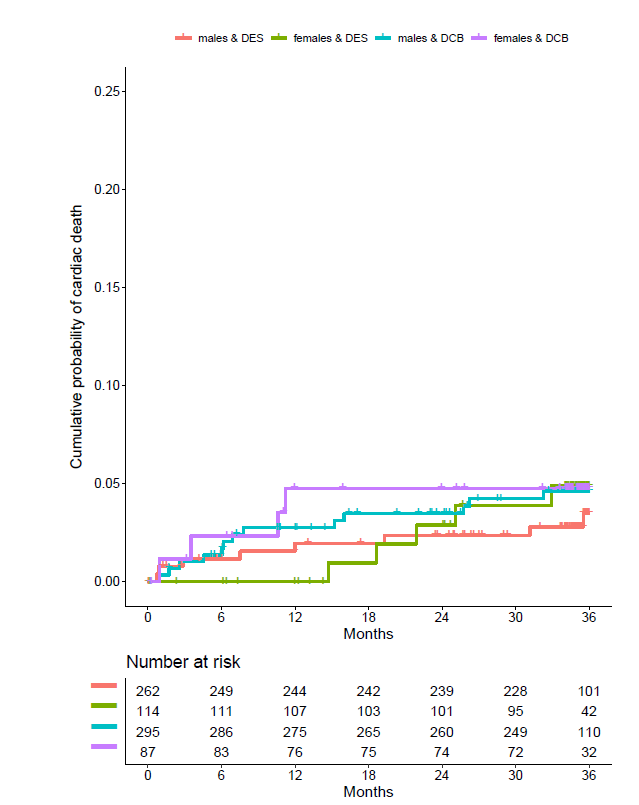


| Figure 1S | Time to cardiac death |
| --- | --- |
| Kaplan-Meier estimates time-to-event curves for cardiac death during 3 years according to sex and PCI strategy | |

**Figure 2S**


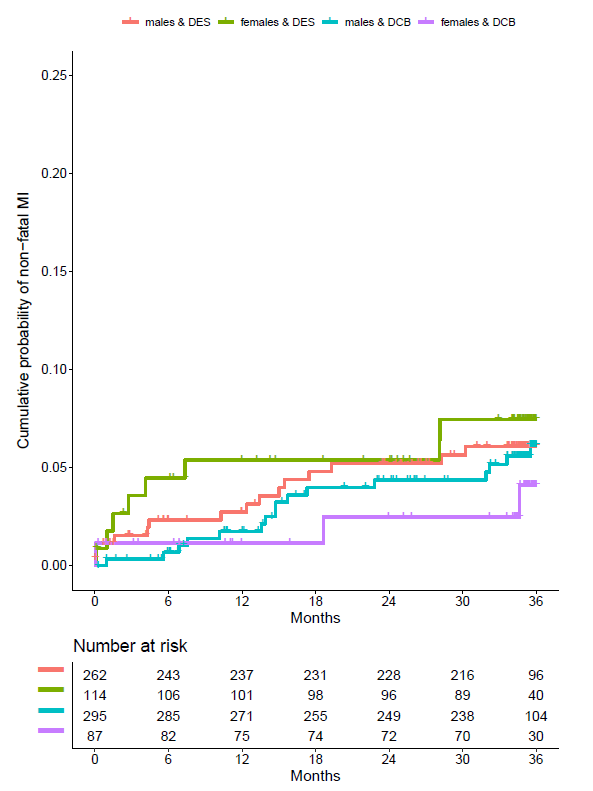


| Figure 2S | Time to non-fatal myocardial infarction |
| --- | --- |
| Kaplan-Meier estimates time-to-event curves for myocardial infarction during 3 years according to sex and PCI strategy | |

**Figure 3S**


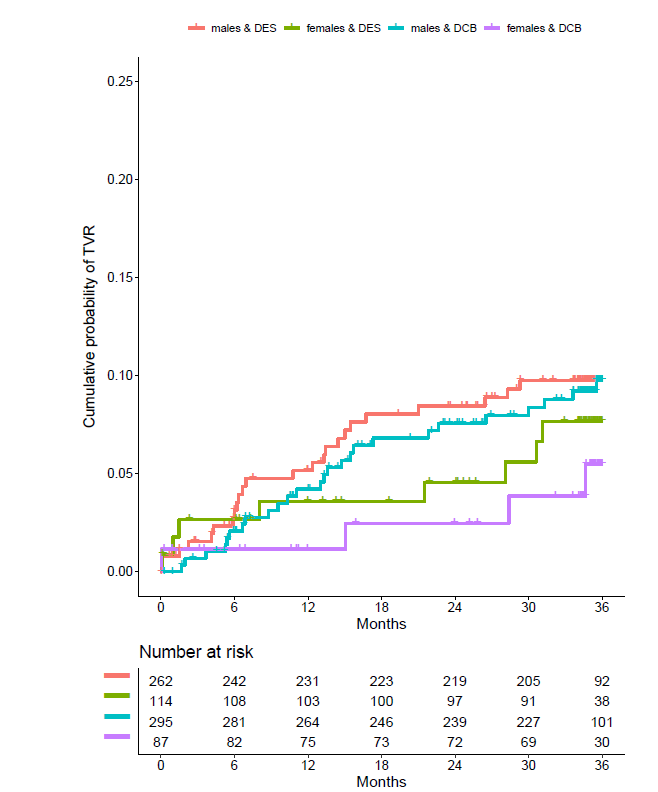


| Figure 3S | Time to target-vessel revascularization |
| --- | --- |
| Kaplan-Meier estimates time-to-event curves for target-vessel revascularization during 3 years according to sex and PCI strategy | |
